# 

**Published:** 1886-10-09

**Authors:** 


					CONTENTS.
Notices- - - - - - - ' 17
well Done !
Discharged.
By George Fleming.
Chapters II and III
Hospital Worthies.
Samuel Morley
Words of Consolation
Notes and News
I he Editor's Letter Box -
Annotations -
Flowers, Ferns, and Ward Decorations
Difficult Cases
Till the Doctor Comes
Prize Competitions -
The Hospital Charity Box -
The Children's Corner
Column for the Curious
The Out-Patients' Diary
PUBLISHED AT
THE OFFICE OF "THE HOSPITAL," 30 & 32, SARDINIA STREET, LINCOLN'S INN FIELDS.
To be had of all Booksellers and Newsagents in the United Kingdom, and at all the
Railway Bookstalls of Messrs. W. H. Smith & Son.

				

